# Fault Tolerant Spectral/Spatial Optical Code Division Multiple Access Passive Optical Network

**DOI:** 10.3390/s24227355

**Published:** 2024-11-18

**Authors:** Rahat Ullah, Sibghat Ullah, Jianxin Ren, Yaya Mao, Zhipeng Qi, Jamil Hussain, Feng Wang, Faheem Khan, Waqas Ahmed Imtiaz

**Affiliations:** 1 Institute of Optics and Electronics, Nanjing University of Information Science & Technology, Nanjing 210044, China; rahat@nuist.edu.cn (R.U.);; 2 Jiangsu Key Laboratory for Optoelectronic Detection of Atmosphere and Ocean, Nanjing University of Information Science & Technology, Nanjing 210044, China; 3 Jiangsu International Joint Laboratory on Meteorological Photonics and Optoelectronic Detection, Nanjing University of Information Science & Technology, Nanjing 210044, China; 4 School of Electronic Science and Technology, Southeast University China, Nanjing 210018, China; sibghat@bupt.edu.cn; 5 Department of Artificial Intelligence Data Science, Sejong University, Seoul 05006, Republic of Korea; 6 Department of Computer Engineering, Gachon University, Seongnam-si 13120, Republic of Korea; 7 Electrical Engineering Department, University of Engineering and Technology, Peshawar 25000, Pakistan

**Keywords:** spectral–spatial, optical code division multiple access (OCDMA), passive optical network (PON), network optimization, two dimensional (2D)

## Abstract

High-capacity communication networks are built to provide high throughput and low latency to accommodate the growing demand for bandwidth. However, the provision of these features is subject to a robust underlying network, which can provide high capacity with maximum reliability in terms of the system’s connection availability. This work optimizes an existing 2D spectral–spatial optical code division multiple access (OCDMA) passive optical network (PON) to maximize connection availability while maintaining desirable communication capacity and capital expenditure. Optimization is performed by employing ring topology at the feeder level, which is used to provide a redundant path in case of connection failures. Furthermore, high transmission capacity is ensured by utilizing a pseudo-3D double-weight zero cross-correlation (DW-ZCC) code. The analysis is performed with Optisystem simulations to observe the performance of the system in terms of bit error rate (BER), received power, and eye openings. It is observed that the introduction of ring topology at the feeder level of the PON does not impact the overall transmission capacity of the system. The system can still support maximum transmission capacity at receiver sensitivities of up to −19 dB. Reliability analysis also shows that the optimized ring-based architecture can provide desirable connection availability compared to the existing system.

## 1. Introduction

Optical code division multiple access (OCDMA)-based passive optical networks (PONs) have gained significant attention over the past two decades due to their support for asynchronous connectivity with relatively simple architectures. OCDMA-based PONs rely on the principle that each user can access the medium simultaneously through the allocation of a unique code. Each code in the OCDMA family is designed with three basic performance parameters called code length (L), code weight (w), and cross-correlation $\left(\lambda_{c}\right)$, respectively [1,2,3,4].

Communication networks have seen exponential growth in terms of data and the number of subscribers over the past few decades, and with the introduction of networks like beyond 5th generation (B5G) wireless communication technology, 6th generation (6G) networks, and next-generation PONs (NG-PONs), the need for a robust underlying network has become more severe than ever [5,6,7,8,9,10,11,12,13,14,15]. In order to overcome the stringent challenges of high capacity and cardinality along with affordability, OCDMA codes evolved from a one-dimensional (1D) paradigm to two-dimensional (2D), three-dimensional (3D), and pseudo-3D domains. The basic goal of this expansion is to offer the best possible combination of L, w, and $\lambda_{c}$ properties in order to support the ever-growing demand for the upcoming networks [5].

**Figure 1:**
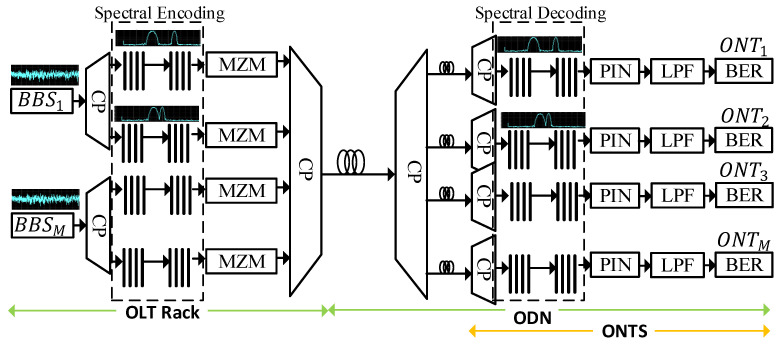
One-dimensional (1D) spectral amplitude coding (SAC) OCDMA architecture.

One-dimensional (1D) OCDMA codes, as the name suggests, utilize code sequences spread along a single dimension to facilitate asynchronous connectivity over the communication channel [1]. Figure 1 represents a basic 1D OCDMA system employing spectral amplitude coding (SAC). Each user is assigned a specific code sequence translated from binary 1s into a spectral representation at the spectral encoding arrangement. A broadband source (BBS) provides the required spectrum, and an optical coupler (CP) splits the BBS spectrum to facilitate multiple subscribers. The encoded spectrum is modulated at a Mach–Zehnder modulator (MZM) and transmitted over the optical distribution networks using an optical combiner [1,2,3].

The receiving optical network terminal (ONT) utilizes a spectral decoding arrangement in accordance with the spectral encoder to receive and recover the intended spectrum. The received spectrum is then converted from the optical to the electrical domain using a PIN photodiode. A low-pass filter (LPF) is employed to perform the required filtering before the signal is analyzed at the bit error rate (BER) module.

The performance of 1D OCDMA systems is primarily translated by N, w, and $\lambda_{c}$ of the coding scheme. Such schemes fail to support high-cardinality systems, owing to the fact that code length increases significantly with an increase in the number of users. Furthermore, the requirement of ideal or fixed in-phase cross-correlation also limits the support of 1D OCDMA systems for high-capacity connectivity. Consequently, 2D and 3D codes were developed to overcome the limitations of 1D code.

Two-dimensional (2D) OCDMA systems employ two coding schemes along the $X^{th}$ and $Y^{th}$ domains to overcome the problem associated with a limited number of codes while maintaining zero or fixed in-phase cross-correlation. Such systems exhibit efficient performance, owing to the fact that they are able to negate the effects of multiple access interference (MAI) and associated phase-induced intensity noise (PIIN) more effectively in comparison to their 1D counterparts. The $X^{th}$ and $Y^{th}$ domains in 2D codes primarily utilize three different techniques, spectral, spatial, or temporal, for the encoding and decoding processes at the transmitter and receiver modules, respectively. On the contrary, 3D codes employ three distinct coding schemes along the $X^{th}$, $Y^{th}$, and $Z^{th}$ domains to fulfil the system requirements [16].

**Figure 2:**
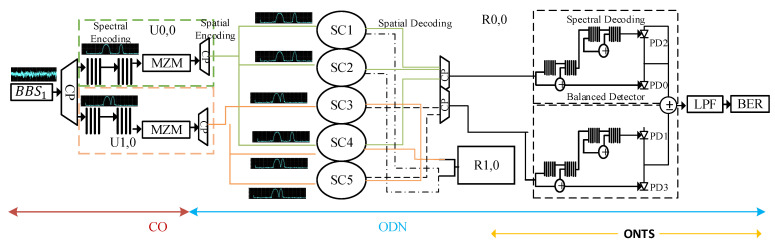
Two-dimensional (2D) spectral–spatial OCDMA architecture.

Spectral–spatial systems have been known as the most effective combination in the 2D OCMDA code family for providing high capacity in terms of data, reach, and the number of subscribers. Such systems utilize spectral conversion of binary codes along the $X^{th}$ domain and spatial encoding and decoding along the $Y^{th}$ domain. Figure 2 shows a conventional spectral–spatial OCDMA system with two users, $U_{0,0}$ and $U_{1,0}$, at the central office (CO) of the network. It also demonstrates the operation of the 2D spectral–spatial OCDMA system, in which spectral encoding is implemented at the first phase of the network with the help of a BBS, coupler (CP), and specifically configured filtering arrangement. User data are modulated after spectral encoding with the help of MZM [16,17,18,19,20].

The end-face of the MZM is fed to a spatial encoding arrangement that starts by splitting the incoming signal into three equal parts depending on the weight of the OCDMA code. The equally distributed spectral–spatial encoded signal is transmitted over the ODN via long-span parallel optical fiber paths. The light wave paths are then received by the corresponding star couplers (SCs) at the remote (RN). The selection of a corresponding SC depends on the OCDMA code. The output ports of the SCs are fed to distribution fibers that transmit the signal towards the ONT module.

The ONT module employs a balanced detector arrangement to receive the incoming spatially decoded signal. The balanced detector is utilized to recover the intended spectrum through spectral decoding. The decoded signal is then passed through an LPF and BER module to retrieve the transmitted information. It can be observed that a two-user spectral–spatial OCDMA system with OCDMA code of w=3 and $\lambda_{c}=1$ requires a total of sixteen long-span optical fiber paths and five star couplers ($SC_{1}-SC_{5})$.

Several combinations have been developed to implement high-capacity 2D OCDMA systems. Ref. [17] presents a spectral–spatial OCDMA system using zero cross-correlation codes and multi-mode fibers (MMF). The primary purpose of employing MMF is to reduce the overall number of optical fiber (OF) strands at the optical distribution network. However, MMF are not able to support long-reach connectivity. Furthermore, ZCC code uses code sequences with w=3, which places a cap on the total number of users. The authors in [18] have proposed a 2D spectral–spatial OCDMA system for free space optical (FSO) communication while using a new two-dimensional successive weight (2D-SW) code. However, no solution is provided to reduce the number of fiber strands at the ODN level. A hybrid OCDMA/OFDM system is proposed in [19] to achieve a high data rate at extended reach and a relatively large number of subscribers. The solution can extend the system’s communication capacity; however, no work is conducted toward reducing fiber strands at the feeder level. A different study in [20] uses the polarization approach to increase the system’s transmission capacity. However, the polarization technique does not compress the OCDMA codes, which makes it difficult to achieve large cardinality.

Three-dimensional OCDMA codes have been proposed to further elevate the cardinality and capacity of the communication system by reducing the overall complexity of the OCDMA network. Ref. [21] proposes a 3D OCDMA architecture based on MD with spectral, temporal, and spatial encoding. The proposed architecture claims to reduce the overall system complexity by 50 percent and offers acceptable transmission capacity. However, no remedy is provided for the reduction in multiple parallel paths along the feeder level to implement spatial encoding. Reference [22] proposes a 3D OCDMA system based on multi-service (MS) and multi-diagonal (MD) OCDMA codes while using spectral–temporal and spatial encoding. MS code offers fixed in-phase cross-correlation between the adjacent codes because balanced detection is employed at the receiving end.

Furthermore, spatial encoding is performed with multiple parallel optical fiber media, which reduces the feasibility of system implementation in terms of cost and complexity. A three-dimensional code with zero cross-correlation property has been proposed in [23]. However, the temporal encoding is employed before the spatial encoding arrangement, and no mechanism is utilized to reduce the number of long-span parallel optical fiber paths between the transmitter and receiver modules. A new three-dimensional successive weight (3D-SW) code OCDMA system has been proposed in [7] for FSO application. The proposed systems offer zero cross-correlation between the adjacent codes. However, employment of conventional spatial encoding results in the utilization of several FSO links.

It can be observed from the aforementioned discussion that 1D OCDMA systems are not able to fulfill the ever-growing demand for high-capacity connectivity due to their limited support for a large number of subscribers. Therefore, 2D spectral–spatial OCDMA codes were proposed to offer a large number of codes at acceptable code lengths and cross-correlation properties. However, the utilization of spatial encoding complicates the overall implementation of the system due to its inherent requirement for a large number of parallel optical fiber paths. Three-dimensional (3D) OCDMA systems utilizing spectral, spatial, and temporal encoding techniques were proposed to overcome the limitations of 2D systems regarding data, reach, and the number of subscribers.

Nevertheless, no action has been taken to mitigate the problem associated with deploying parallel optical fiber paths for spatial encoding that increases overall complexity and cost of the system and reduces its feasibility of deployment despite high-capacity connectivity. The problems mentioned above have been solved in [5] by implementing a pseudo-3D OCDMA system that employs temporal encoding after the spatial encoding process to reduce multiple parallel optical fiber paths to a single fiber. However, no protection is provided at the feeder level, which decreases the reliability and associated connection availability of the system.

To develop an OCDMA architecture that can provide high transmission capacity and acceptable connection availability, this work optimizes the tree-based architecture proposed in [5]. Zero cross-correlation code and the employment of temporal encoding outside the transmitter module facilitate the mitigation of spatial multiplexing, enabling paths over the distribution network (ODN). This is achieved by utilizing a pseudo-3D OCDMA code that employs time delay units (TDUs) after the spatial encoding module to overcome the problem of spectrum overlap over a single communication channel. The proposed model minimizes the network’s overall cost and also provides high capacity in terms of data, reach, and the number of subscribers.

Furthermore, ring-based topology is deployed at the feeder level to overcome the problem of a single point of failure associated with the tree-based architecture. The analysis of the proposed architecture is performed in two stages. The first stage of the analysis evaluates the system’s transmission capacity by implementing the proposed model in Optisystem and observing bit error rate (BER) and eye diagrams against the length of optical fiber media deployed between the remote nodes. The second stage of the analysis observes the improvement in connection availability by performing a reliability analysis in comparison with the conventional structure proposed in [5].

## 2. Proposed Architecture

The proposed architecture is developed by making changes at the OLT and feeder level of the 2D spectral–spatial OCDMA-based PON in [5]. A switching arrangement is introduced at the transmitter module to facilitate traffic switching between clockwise and counterclockwise flow in the case of failures at the feeder level. Furthermore, a conventional tree-based setup is replaced with a ring-based topology to provide desirable connection availability.

The transmitter module starts with multiple broadband light sources (BBSs) that are utilized to provide the required spectrum for OCDMA encoding and transmission of the electrical pulses over the optical fiber (OF) medium. The OCDMA code determines the number of BBSs employed at the transmitter module. The proposed architecture is deployed with 2D double-weight zero cross-correlation (2D DW-ZCC) code that is developed with 1D DW-ZCC code sequences employed at the spectral $\left(X^{th}\right)$ and spatial $\left(Y^{th}\right)$ domains, respectively [4,5,24]. The 1D DW-ZCC code belongs to the family of SAC-OCDMA codes. It is developed with three basic performance parameters, code length (L=K×w), code weight (w=2), cross-correlation $\left(\lambda_{c}=0\right)$, and auto-correlation $\left(\lambda_{a}=w\right)$. Here, K represents the total number of codes or subscribers in the system [5].

The same parameters are now employed along the spectral and spatial domains to develop a 2D structure of DW-ZCC code shown in Table 1, which gives four 2D DW-ZCC codes as $S_{0,0}={Y_{0}^{T}X}_{0}$, $S_{0,1}={Y_{0}^{T}X}_{1}$, $S_{1,0}={Y_{1}^{T}X}_{0}$, and $S_{1,1}={Y_{1}^{T}X}_{1}$. $S_{0,0}$ is used to represent the first subscriber of the system with a code sequence of $Y_{0}^{T}X_{0}$. Similarly, $S_{0,1}$ is used to represent the second subscriber of the network having a unique code sequence of $Y_{0}^{T}X_{1}$. Here, X is utilized to represent the spectral code sequence, and Y is used to represent information about the spatial code sequences that are utilized to build the 2D DW-ZCC code matrix. It can be observed that user $S_{0,0}$ is allocated a code sequence of $Y_{0}^{T}X_{0}$, with $X_{0}=[1100]$ and $Y_{0}=[1100]$ being the spectral and spatial code sequences, respectively. In general, the code matrix for 2D DW-ZCC code can be written as $Z_{ij}=Y_{j}^{T}X_{i}$, where i=(1,2,3,……,M) and j=(1,2,3,……,N) represent spectral and spatial code sequences. Table 1 represents the basic structure of the 2D DW-ZCC codes built with $X_{0},X_{1},Y_{0}^{T}$, and $Y_{1}^{T}$, respectively [5,25].

The 2D DW-ZCC code is utilized for the development of the proposed architecture because it provides the following superior features in comparison with the existing counter parts:

1. Provision of maximum auto-correlation $\left(\lambda_{a}=w_{1}w_{2}\right)$ and minimum cross-correlation $\left(\lambda_{c}=0\right)$, which can also be expressed as $C^{\left(d\right)}\left(g,h\right)=\sum_{i=1}^{M}\sum_{j=1}^{N}{dz}_{\left(i,j\right)}^{\left(d\right)}{dz}_{\left(i,j\right)}(g,h)$.
2. The adjacent placement of binary chips in both the spectral and spatial code sequences significantly simplifies the design of the spectral encoder and decoder modules through the utilization of a single filtering arrangement of binary to spectral encoding and vice versa.
3. The existence of zero cross-correlation between all codes of the matrix facilitates the use of the spectral direct detection (SDD) technique, which is known to yield efficient performance in comparison with the existing counterparts by completely eliminating MAI and associated PIIN.

**Figure 3:**
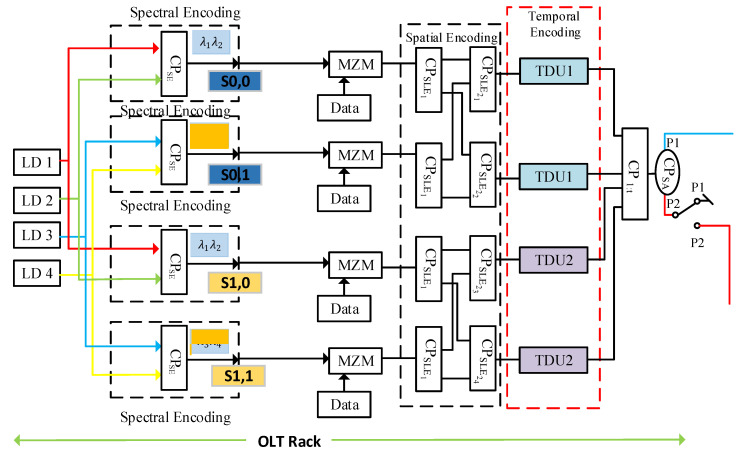
Transmitter section for optimized 2D DW-ZCC-based OCDMA architecture.

Figure 3 demonstrates the architecture of the transmitter section for the proposed 2D DW-ZCC OCDMA-based PON with ring topology. The given architecture is designed for M×N subscribers (for demonstration purposes). Here, M=N=2 represents the $X^{th}$ and $Y^{th}$ code sequences shown in Table 1. Four broadband laser diodes (LDs), having wavelengths of $\lambda_{1}$, $\lambda_{2}$, $\lambda_{3}$, and $\lambda_{4}$, are utilized to construct the given architecture. The output of each LD is equally split into two parts using a 1:2 power splitter. LDs provide the required broadband spectrum for carrying the electrical signals over the optical fiber media. Furthermore, the output of the LDs is encoded with the help of the spectral encoder in order to translate the binary 1s and 0s in the $X^{th}$ code sequence to their required representation in the spectral domain.

Now, for the subscriber $S_{0,0}$, utilizing the spectral code sequence $X_{0}=[1\;1\;0\;0]$, the outputs from LD 1 and LD 2 having wavelengths of $\lambda_{1}$ and $\lambda_{2}$ are fed to a 2:1 power coupler $\left(CP_{SE}\right).$ Here, CP is used to represent the power coupler, and the subscript SE is used to specify the spectral encoding operation. Thus, $CP_{SE}$ is a spectral encoder that uses the power combiner to encode the intended spectrum from the binary into the spectral domain as per the $X^{th}$ code sequence. The end-face of the PC is fed to a Mach–Zehnder modulator (MZM), as shown in Figure 3. The MZM performs the required electrical-to-optical modulation through an on–off keying (OOK) operation. The OOK operation involves the conversion of user data from the electrical to optical domain by combining it with the encoded spectrum of $\lambda_{1}$ and $\lambda_{2}$.

The next phase of the encoding process includes spatial encoding that is performed by splitting the incoming spectrally encoded signals from the MZM into multiple parallel paths of w=2 using a 1:2 power coupler $\left(CP_{SLE_{1}}\right)$. Here, CP represents the power coupling operation, whereas the subscript SLE is used to specify the spatial encoding operation. The spatial encoding, unlike the conventional spectral–spatial OCDMA system, consists of two steps. The first step of the encoding process is represented with the subscript $SLE_{1}$, followed by the second step components represented with $SLE_{2}$, respectively. For the first stage of the spatial encoding, the end-face of the MZM is fed to a 1:2 splitter, which is used to split the incoming signal into equal portions, respectively. The splitting ratio of the $CP_{SLE_{1}}$ is defined by the weight of the $Y^{th}$ code sequence. The splitting operation performed by ${CP}_{SLE_{1}}$ essentially splits the spectrally encoded signal into multiple parallel paths as per the requirement of the spatial encoding operation.

The next step of the spatial encoding process involves the combination of the encoding signals from the output ports of the 1:2 $CP_{SLE_{1}}$ to their corresponding power combiners implemented by using a 2:1 power coupler $\left(CP_{SLE_{2}}\right)$. Here, $SLE_{2}$ is used to implement the second phase of the spatial encoding process. $CP_{SLE_{2}}$ is used to collect the multiple parallel paths coming out of $CP_{SLE_{1}}$ and combine them in order to overcome the problem associated with long-span optical fiber paths in the conventional spectral–spatial OCDMA systems.

The choice of the $CP_{SLE_{2}}$ is determined by the placement of binary 1s on the $Y^{th}$ code sequence. For instance, for the subscriber $S_{0,0}$, utilizing the spatial code sequence $Y_{0}=\left[1\;1\;0\;0\right]$, $CP_{SLE_{2_{1}}}$ and $CP_{SLE_{2_{2}}}$ are utilized to implement the spatial encoder. Similarly, for the subscriber $S_{1,0}$, utilizing the spatial code sequence $Y_{0}=\left[0\;0\;1\;1\right]$, $CP_{SLE_{2_{3}}}$ and $CP_{SLE_{2_{4}}}$ shall be used. Spectral power couplers coupled with power splitters at the spectral decoder are employed in this architecture to replace the use of a star coupler (SC), shown in Figure 2*,* that requires several optical fiber paths parallelly employed to enable spatial encoding and decoding.

Time delay units (TDUs) are introduced to perform temporal encoding with the $Y^{th}$ code sequence. Furthermore, TDUs are employed to further reduce the optical fiber paths coming out of the ${CP}_{SLE_{2}}$ power combiners by delaying the signals through a specific time period so that they can be combined and transmitted over a single optical fiber path across the feeder level (FL). Now, for the subscriber $S_{0,0}$, output legs of $CP_{SLE_{2_{1}}}$ and $CP_{SLE_{2_{2}}}$ are fed into time delays units ${TDU}_{1}$ and ${TDU}_{2}$, respectively. Similarly, for the subscriber $S_{1,0}$, utilizing the spatial code sequence $Y_{0}=\left[0\;0\;1\;1\right]$, ${CP}_{SLE_{2_{3}}}$ and ${CP}_{SLE_{2_{4}}}$ outputs shall be fed to the time delays units ${TDU}_{3}$ and ${TDU}_{4}$, respectively. The end-faces of the temporal encoders are then fed into a 1:t power combiner ${CP}_{1:t}$, where t represents the total number of TDUs utilized at CO. The power combiner ${CP}_{1:t}$ is utilized to collect all the encoded signals and combine them for transmission over a single mode optical fiber (SMF) medium.

Optimization in the existing 2D DW-ZCC OCDMA-based PON is performed at the transmitter module by utilizing a switching arrangement. This arrangement is employed to facilitate a clockwise flow of traffic under normal operating conditions and switch traffic flow between clockwise and counterclockwise under failures at the FL. The switching arrangement’s anatomy is shown in Figure 3. The switching arrangement is developed with a 1:2 power coupler ${CP}_{SA}$. The main function of ${CP}_{SA}$ is to split the encoded spectrum into two equal parts in order to facilitate the flow of traffic in clockwise and counterclockwise directions for implementation of the ring architecture at the feeder level.

**Figure 4:**
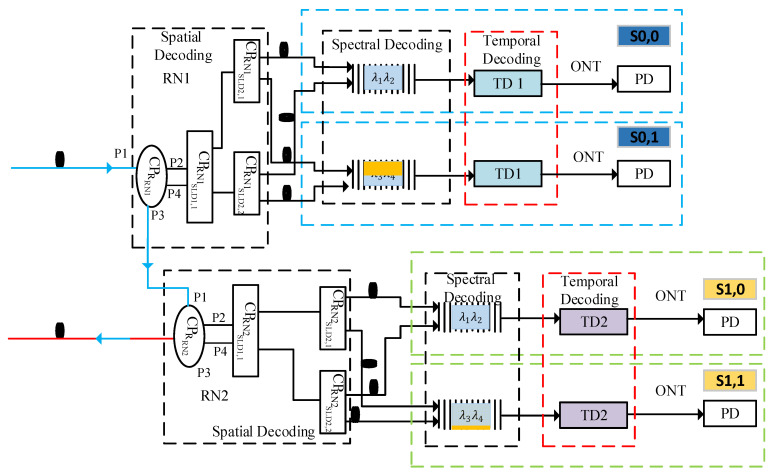
ODN and receiver section of the proposed 2D DW-ZCCC ODCMA architecture.

Output port 1 of the ${CP}_{SA}$ is utilized to carry the traffic in a clockwise direction under normal operating conditions. Port 2 of the ${CP}_{SA}$ is connected to a switch, ${SW}_{SA}$, as shown in Figure 2. The switching arrangement ${SW}_{SA}$ is utilized to control the flow of traffic under normal operating conditions and in the event of failure. Port 1 of the switch is normally grounded to halt traffic flowing in a counterclockwise direction under normal operating conditions. However, in the event of failure at the FL, the switch moves from port 1 towards port 2. This feature enables the flow of traffic in both a clockwise direction through ${SW}_{SA}$ port 1 and a counterclockwise direction through ${SW}_{SA}$ port 2, respectively. The end-face of the switching arrangement is fed to the feeder-level ring-based fiber as shown in Figure 4.

The ring-based fiber deployed along the FL carrying traffic in the clockwise direction is received by the first remote node $\left(RN_{1}\right)$, being the first node at the feeder level. The feeder fiber carrying traffic in the clockwise direction is referred to as $\left(FF_{cw}\right)$. Similarly, the second half of the ring carrying traffic in the counterclockwise direction is collected by the second remote node $\left(RN_{2}\right)$, being the last node at the feeder level; this section of the fiber is called $\left(FF_{cCw}\right)$.

The internal structure of $RN_{1}$ is shown in Figure 4, which essentially houses multiple passive couplers to facilitate the formation of the ring at the feeder level along with the establishment of spatial decoding for the distribution level [25,26,27]. The $FF_{cw}$ is received by a 2:2 optical coupler $\left(C{P_{R}}_{RN_{1}}\right).$ Here, ${CP}_{R}$ is used to represent the coupler that is employed for the formation of the ring topology at the feeder level and extension of traffic towards the feeder level. Furthermore, the subscript $RN_{1}$ represents the first remote node employed across the ring topology.

Ports 1 and 3 of the $C{P_{R}}_{RN_{1}}$ are utilized for formation of the ring at the feeder level, such that $C{P_{R}}_{RN_{1}}$ receives the incoming traffic through port 1 and extends it towards the feeder level via port 3. Ports 2 and 4 of $C{P_{R}}_{RN_{1}}$ are extended towards a 1:2 optical coupler $\left(C{P_{RN1}}_{SLD_{1,1}}\right)$, as shown in Figure 4, and are used to transfer the received encoded spectrum towards the ONT module through the distribution level [25,26].

The optical coupler $C{P_{RN1}}_{SLD_{1,1}}$ is used as the first stage of the spatial decoding process. The basic purpose of $C{P_{RN1}}_{SLD_{1,1}}$ is to split the incoming signal into equal parts as per the weight of the $Y^{th}$ code sequence. Here, ${CP}_{RN1}$ represents the coupler employed at the first RN, and $SLD_{1,1}$ represents the first coupler employed at the first RN for the spatial decoding operation. Both legs of the $C{P_{RN1}}_{SLD_{1,1}}$ are connected to two 1:2 optical couplers: $\left(C{P_{RN1}}_{SLD_{2,1}}\right)$ and $\left(C{P_{RN1}}_{SLD_{2,2}}\right)$, respectively. Here, $SLD_{2,2}$ represents the first coupler that is employed to perform the second phase of the spatial decoding operation. This phase consists of splitting the signal into two equal parts so that each node at the receiving end receives its intended spectrum from multiple parallel paths. This is achieved by connecting both couplers to the distribution fibers that are extended towards the ONT modules.

At the receiving ONT, for subscriber $S_{0,0}$, the distribution fibers are received by a filtering arrangement, which essentially consists of band-pass filters configured to recover the intended spectrum, as shown in Figure 4. The reception arrangement is configured such that the user $S_{0,0}$ receives one DF from $C{P_{RN1}}_{SLD_{2,1}}$ and another from the $C{P_{RN1}}_{SLD_{2,2}}$, respectively. This is performed to accomplish the process of spatial decoding as per the encoding arrangement performed at the transmitter module. Furthermore, the setup is configured in a way to ensure the recovery of $\lambda_{1}$ and $\lambda_{2}$ for $S_{0,0}$.

The output of the spatial decoder is then fed to the temporal decoder that is configured in accordance with ${TDU}_{1}$ to perform the required temporal decoding at the ONT module. The decoded signal is then applied to the photodiode, which converts the optical signal into the electrical domain for necessary filtering and recovery operation.

## 3. Performance Analysis

This section analyzes the performance of the proposed architecture in terms of bit error rate (BER) and eye diagrams. The analysis is performed for a total of 128 subscribers in Optisystem. The proposed model is implemented with 32 $X^{th}$ code sequences and 4 $Y^{th}$ code sequences. The given number of subscribers is implemented with 64 LDs that are centered at 191.5 THz, 191.6 THz, and onwards until 197.9 THz. It is pertinent to mention that the utilization of 32 $X^{th}$ code sequences and 4 $Y^{th}$ code sequences enables the implementation of the proposed setup’s broader wavelength spectrum, which also increases the overall spectral efficiency of the system. Moreover, a wider wavelength spectrum minimizes crosstalk and interference between channels. If the wavelength range is too close, it can cause overlap in the frequency domain, leading to increased noise and potential errors in data transmission.

Furthermore, four remote nodes are employed at the feeder level, and each remote node is assigned a total of 32 subscribers. For instance, $RN_{1}$ is assigned the subscribers with spectral code sequences from $X_{0}$ to $X_{31}$ and a spatial code sequence of $Y_{0}^{T}.\;$ Similarly, $RN_{2}$ is assigned the subscribers with spectral code sequences from $X_{0}$ to $X_{31}$ and spatial code sequences ranging from $Y_{1}^{T}.$ Moreover, four time delay instances, from $TDU_{0}$ to $TDU_{3}$, are employed for simulation implementation, with reference of the four $Y^{th}$ code sequences. The performance parameters that have been utilized to perform the given analysis are presented in Table 2.

**Figure 5:**
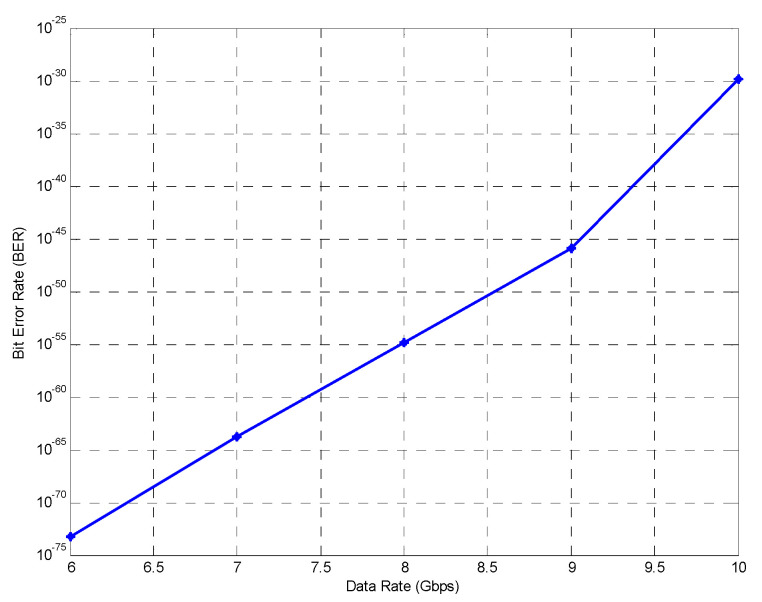
BER vs. data rate for B2B model.

For the preliminary analysis, a back-to-back (B2B) model is utilized, and the BER is measured against different data rates for simultaneously accessing subscribers. It can be observed from Figure 5 that the BER, which is given as a log of the BER, increases with an increase in the amount of data that is being transmitted over the shared medium. As the data rate increases, the bit durations become shorter, which can cause pulse spreading and result in signal overlap, leading to inter-symbol interference. Additionally, the signal power is spread over a larger bandwidth at higher data rates, which can reduce the power spectral density [28]. However, the robust correlation properties of the DW-ZCC code and utilization of the spectral direct detection technique make sure that only the intended spectrum is recovered with maximum auto-correlation and minimum cross-correlation. Consequently, the proposed setup is able to support a data rate of up to 10 Gbps.

The proposed system’s performance is further analyzed by observing its reach in terms of the BER values through varying optical fiber (OF) lengths. The analysis is performed at randomly selected ONT nodes at each RN while 128 users access the medium simultaneously at 10 Gbps of data per subscriber. Moreover, the length of the OF medium is increased by a factor of 1 km between the adjacent RNs for each analysis. The system performance parameters in Table 2 are utilized for this study.

**Figure 6:**
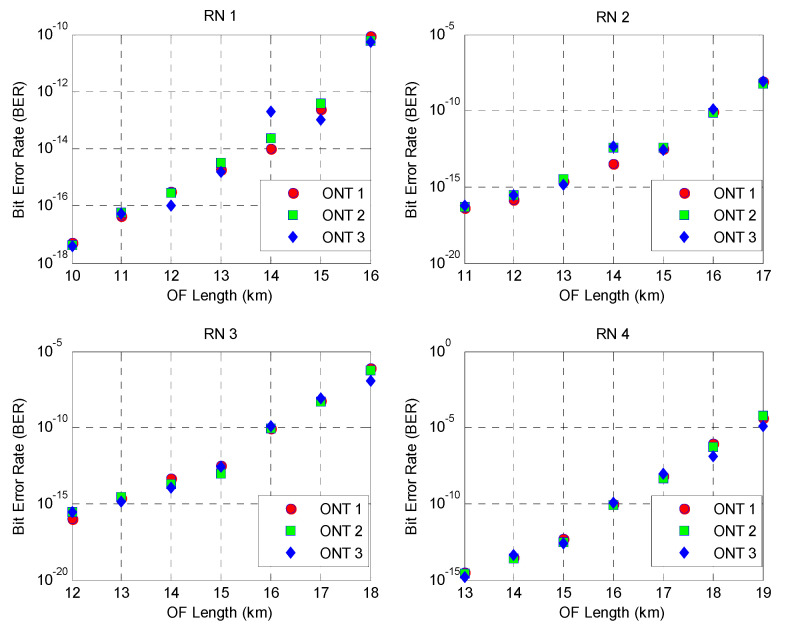
BER at different RNs for varying optical fiber (OF) lengths.

It can be observed from Figure 6 that the proposed system can provide relatively lower values of BER, in the order of $10^{-18}$, at 10 km of the deployed OF media between the transmitter and $RN_{1}$. However, a progressive increase in the BER is observed with growth in the reach of the OF media, demonstrating the cumulative effect of signal degradation. For instance, $ONT_{1}$ nodes at $RN_{1}$ are able to provide BER values of approximately $5.11\times 10^{-13}$ at a fiber length of 10 km, indicating highly reliable transmission. However, as the length of the OF media increases to 16 km, the BER rises to as high as $8.3\times 10^{-11}$. This indicates a drop in the transmission quality.

This can be attributed to the fact that signal attenuation and dispersion increase along the length of the OF media, which increases the signal-to-noise ratio in the receiving photodiode. Correspondingly, the BER values are affected, and an increase in the BER is observed as the length of the OF media increases. Nevertheless, it can be observed from the graph patterns that the proposed 2D DW-ZCC with an efficient combination of spectral–spatial and temporal encoding systems is still able to support high transmission capacity in terms of data, reach, and the number of subscribers, owing to the utilization of the ideal cross-correlation property between adjacent codes.

An analysis of Figure 6 shows a relatively steeper rise in the BER values for the ONT nodes at $RN_{3}$ and $RN_{4}$. For instance, BER values of 3.5 $\times 10^{-15}$ and $4.5\times 10^{-5}$ are observed for ONT1 of RN4 at 13 km and 19 km OF lengths, respectively. This shows a relatively greater decline in the system’s performance, which can be attributed to the fact that the ONT nodes at $RN_{3}$ and $RN_{4}$ are experiencing higher signal attenuation and dispersion along the OF media as compared to the other nodes due to utilization of the ring topology at the feeder level, as the signal needs to traverse across all RNs and underlying OF media to reach its corresponding destination. Nevertheless, it can be observed that, for an acceptable BER value of $10^{-9}$, the proposed system is still able to support 128 subscribers communicating at 10 Gbps of data across the OF span of 16~17 km, respectively.

**Figure 7:**
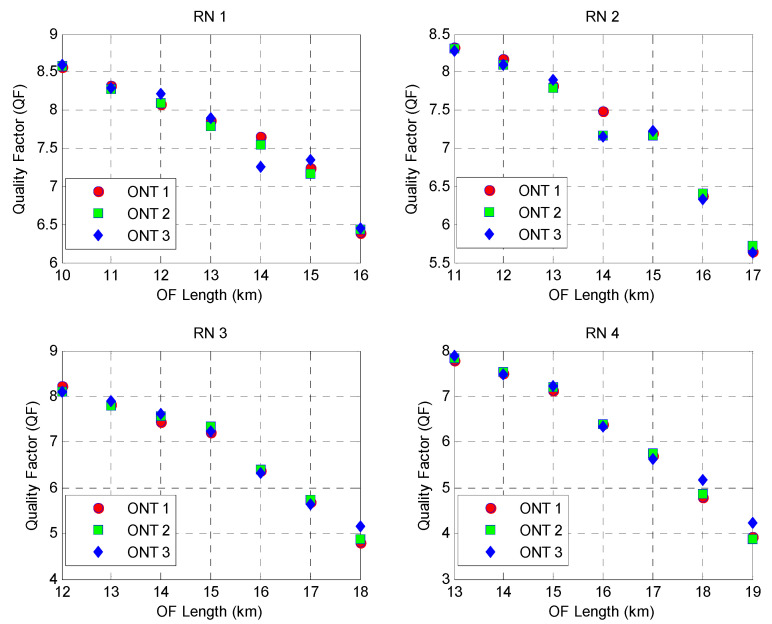
QF at different RNs for varying optical fiber (OF) lengths.

Figure 7 demonstrates the Q-factor (QF) at various ONT nodes of each RN for the proposed 2D DW-ZCC system with spectral–spatial and temporal encoding. The graph trends in Figure 7 demonstrate a decline in the QF with an increase in the length of the OF media. This can be attributed to an increase in the BER values at a longer reach of the OF media, owing to the reduction in power of the received signal.

**Figure 8:**
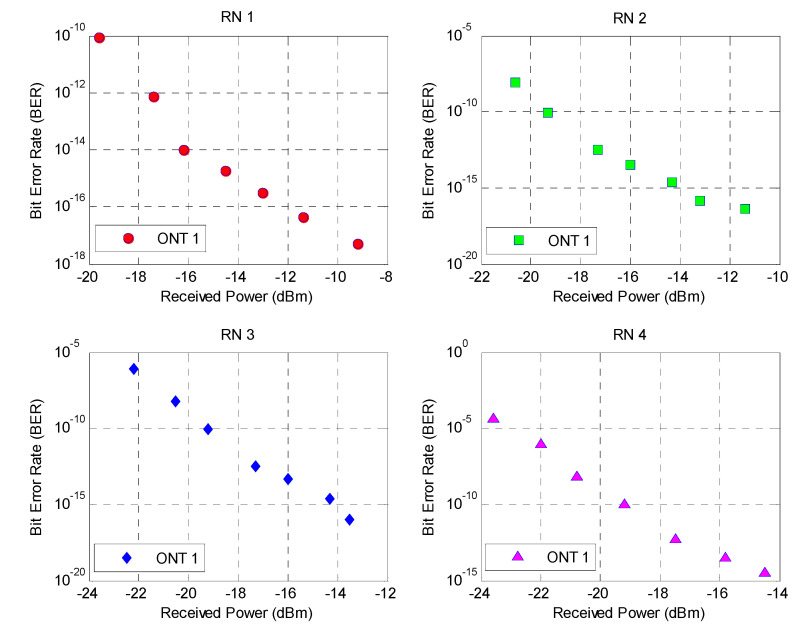
BER versus received power at different lengths of the OF media.

Figure 8 represents the analysis of the proposed system in terms of the BER against the received power at ${ONT}_{1}$ of each RN. Since all ONTs exhibit relatively close BER values, a single ONT module is selected to facilitate clear visualization of the BER versus received power trends. It can be observed that a BER of approximately 5.11 $\times 10^{-18}$ is observed at the ${ONT}_{1}$ modules of ${RN}_{1}$ at a received power of –9.2 dBm for an OF length of 10 km. However, as the length of the OF media increases, the power received at the PIN photodiode decreases, resulting in a BER value of 8.3 $\times 10^{-11}$, and the received power decreases to −19.4 dBm, highlighting a significant reduction in signal integrity. This can be attributed to the fact that the signal-to-noise ratio decreases with a reduction in the received power, which in turn elevates the BER.

A similar trend is observed for ${ONT}_{1}$ of the ${RN}_{2}$ module, where the BER increases from $4\times 10^{-17}$ at −11.4 dBm to $8.5\times 10^{-9}$ at −20.6 dBm, indicating the system becomes more error prone as the power decreases. In the case of the ONT nodes at ${RN}_{3}$ and ${RN}_{4}$, the impact is even more pronounced, with the BER reaching below the acceptable range at values of received power in the range of −19.4~−20.6 dBm, respectively. Furthermore, it can be observed that the signal quality deteriorates rapidly beyond the acceptable range, and a slight decrease in the received power results in relatively large values of BER. Consequently, it can be concluded that the proposed system is able to provide an acceptable BER at the received powers of approximately −19.4 dBm. Beyond the said limit, the proposed system is not able to maintain signal integrity for an acceptable BER value of $10^{-9}$.

Table 3 shows the performance of the proposed system in terms of eye diagrams. The analysis presented in Figure 6 through Figure 8 is utilized to extract the eye diagrams at different operation points for ONT1 of each RN. It can be observed in Table 3 that, as we move from RN1 towards RN3, the height of the eye tends to decrease, indicating a deterioration in signal quality and overall performance of the system. This can be attributed to the fact that the encoded signal has to pass across more splitters and OF media along the proposed ring-based feeder networks, which increases the overall dispersion and attenuation of the signal across the network. This increases dispersion and attenuation and deteriorates the signal-to-noise ratio that, in turn, affects the overall BER values. Consequently, an increase in the BER is observed as the signal moves deep into the network.

The desirable performance delivery of the proposed architecture can be attributed to several key factors of the quasi-three-dimensional DW-ZCC OCDMA code. The code renders ideal correlation properties because the receiving node is able to recover the intended spectrum with maximum power units of $w_{1}$ × $w_{2}$. Similarly, all interfering signals are canceled out completely, owing to the zero cross-correlation property of the DW-ZCC code and utilization of the SDD technique. These features enable the complete recovery of the intended signal with a maximum signal-to-noise ratio. Consequently, performance degradation factors like interference from multiple subscribers are nullified along with a reduction in the inherent noise sources at the receiving photodiode [4,5].

Another factor that is contributing towards the desirable BER values is the overall length of the OCDMA code. A large cardinality matrix of the DW-ZCC code is developed by employing a quasi-three-dimensional approach that is able to support a significantly greater number of users as compared to the previous 1D and 2D versions. Furthermore, PIIN and short noise that are proportional to the length of the code are also mitigated at the receiving end, which enables the attainment of desirable BER values for subscribers communicating simultaneously at 10 Gbps over the shared medium.

## 4. Failure Recovery Analysis

**Figure 9:**
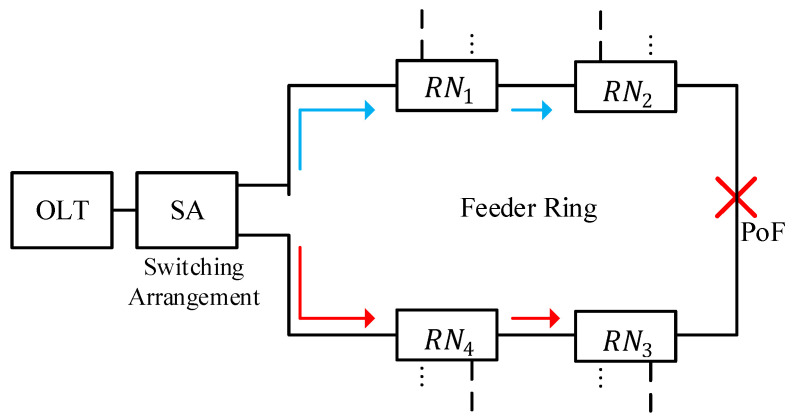
Simulation setup for failure recovery analysis (blue lines show clockwise flow of traffic, red lines show counterclockwise flow of traffic after failure at point of failure (PoF).

This section analyzes the performance of the ring topology in terms of system recovery in the case of failure across the feeder level. Figure 9 represents the arrangement of the simulation setup, where a failure, a fiber cut, is introduced at a certain point of failure (PoF) between $RN_{2}$ and $RN_{4}$ in order to disrupt the conventional flow of clockwise traffic towards all ONT modules of remote nodes from $RN_{1}$ to $RN_{4}$. The switch, ${SW}_{SA}$, employed at the switching arrangement is then flipped from port 1 towards port 2 in order to allow for the flow of traffic in both clockwise and counterclockwise directions as per the system’s description in Section 2.

The BER is observed at ONT 1 of each RN while performing simulation analysis as per the Table 2 performance parameters. It can be observed in Table 4 that the proposed system is able to handle the flow of traffic in both clockwise and counterclockwise directions, as acceptable values of BER are obtained at each ONT module after fiber failure across the feeder level. A degradation in the BER and eye height values is observed as the signal progresses from RN1 to RN2 while receiving clockwise traffic and from RN4 to RN3 for counterclockwise traffic. This behavior aligns with the performance analysis presented earlier, confirming the robustness of the implemented model. Additionally, the results demonstrate that the proposed 2D DW-ZCC system, incorporating spectral–spatial and temporal decoding, can effectively support 128 subscribers in both normal and failure scenarios.

## 5. Reliability Analysis

The convergence of wireless and optical communication technologies has become one of the significant steps in facilitating the 6G network infrastructure. Higher throughput and cardinality, lower latencies, substantially high data rates, and reliability have become the fundamental characteristics of future communication networks. Consequently, this section will analyze the reliability of the proposed ring-based architecture in comparison with the tree-based counterpart proposed in [5]. The reliability analysis is performed in terms of connection availability that can be expressed as follows [20,21,22,23,24,25,26,27,28,29,30]:

(1) $$CA=1-\sum_{i=1}^{j}{UA}_{i}$$

CA in Equation (1) represents the connection availability of the entire network, and $UA_{i}$ is used to give the unavailability of the system components i inside the network. Accordingly, the proposed architecture can be divided into the following blocks to analyze the connection availability of the entire network:

(2) $$UA_{i}={UA}_{CO}+{UA}_{FF}\;+UA_{RNs}+UA_{DF}+UA_{ONTs}$$

Here, CO is used to represent the central office of the network, and ${UA}_{CO}$ refers to the overall unavailability of the components employed at the CO. The same can be further elaborated as follows:

(3) $${UA}_{CO}=UA_{OLT}+UA_{2:1CP_{SE}}+UA_{1:2CP_{SLE_{1}}}+UA_{2:1CP_{SLE_{2}}}+UA_{TDU}+UA_{1:tCP}+UA_{CP_{SA}}+UA_{SW_{SA}}$$

Here, $UA_{OLT}$ gives the unavailability of the system components that are employed at the OLT module, including laser diodes, modulators, filters, data sources, etc. The unavailability of the coupler that is employed to implement the spatial encoding operation is given as $UA_{2:1CP_{SE}}$. Similarly, $UA_{1:2CP_{SLE_{1}}}\;and\;UA_{2:1CP_{SLE_{2}}}$ are used to represent the total unavailability over a given span for the couplers utilized in spatial encoding at the CO. $UA_{TDU},\;and\;UA_{1:tCP}$ are used to give the unavailability of the time delay units and 1:t optical coupler that is used to combine all signals for the feeder level. Switching arrangement components unavailability figures are given as $UA_{CP_{SA}}\;\mathrm{and}\;UA_{SW_{SA}}$, respectively.

Now, the unavailability of the feeder fiber ${UA}_{FF}$ can be expressed as follows:

(4) $${UA}_{FF}={\left({D_{FF}UA}_{OF}\right)}^{2}$$

Here, $D_{FF}$ is used to represent the overall span of the OF medium deployed along the feeder level. ${UA}_{OF}$ is used to employ the unavailability factor of the OF medium. The superscript 2 is utilized in the equation to incorporate the redundant feature of the ring-based topology that provides protection against failures or cuts at the feeder level.

The unavailability of the components employed at the RNs can be expressed as follows:

(5) $$UA_{RNs}=UA_{CP_{R_{RN}}}+UA_{2:2CP_{RN_{SLD1,1}}}+UA_{1:2CP_{RN_{SLD2,1}}}+UA_{1:2CP_{RN_{SLD2,2}}}$$

It can be observed from Figure 3 that each remote node employed four optical couplers. $CP_{R_{RN}}$ is used to represent the first coupler of the RN that receives the incoming signal and splits it for distribution towards the feeder level ring and ONTs at the distribution level. The unavailability of this coupler is represented as $UA_{CP_{R_{RN}}}$. $UA_{2:2CP_{RN_{SLD1,1}}}$ is used to represent the unavailability of the first coupler and is used to initiate the spatial decoding process at the RN. Whereas $UA_{1:2CP_{RN_{SLD2,1}}}\;and\;UA_{1:2CP_{RN_{SLD2,2}}}$ are used to give the unavailability of the coupler employed at the second stage of the spatial decoding process as per Figure 3.

$UA_{DF}$ and $UA_{ONTs}$ are used to represent the unavailability of the OF medium employed at the distribution level and ONT modules, respectively. Mathematically, they can be expressed as follows:

(6) $${UA}_{DF}={D_{DF}UA}_{OF}$$

(7) $$UA_{ONT_{Total}}=UA_{FIL_{SE}}+UA_{TDU}+UA_{ONT}$$

**Figure 10:**
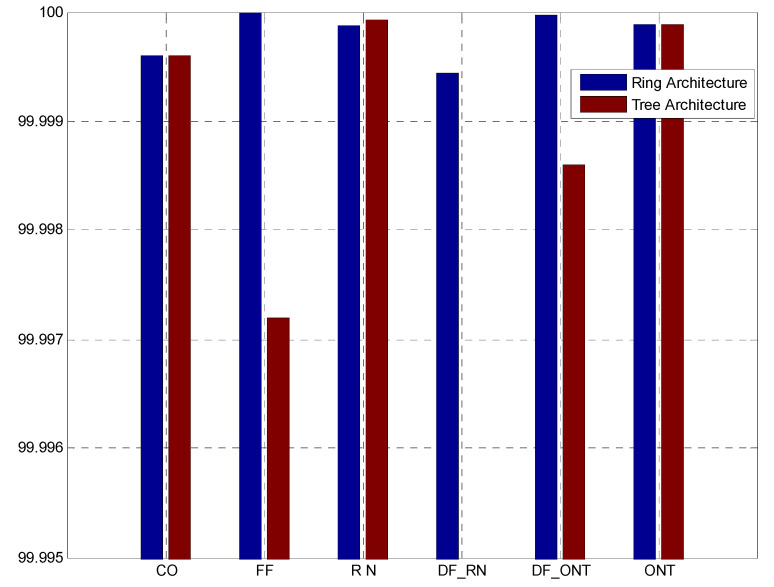
Connection availability of different system components for the proposed and conventional setups.

Figure 10 shows the connection availability of the network components for the tree-based architecture given in [5] against the proposed ring-based network. The given analysis is conducted for the system parameters given in Table 5, and the length of the OF medium is considered as 10 km for the feeder level in both the tree- and ring-based proposed architectures. Furthermore, 2 km OF medium is considered to be deployed between the RNs of the ring topology in the proposed architecture. At the distribution level, 3 km and 5 km OF media are considered for the proposed and conventional architectures, respectively.

The analysis of the connection availability of the different system components demonstrates promising results for the proposed ring-based architecture. It can be observed that the availability of the components housed inside the CO, RN, and ONT modules is well above the acceptable range of 99.999% for both networks.

Figure 10 shows a significant difference between the connection availability readings at the feeder level and distribution level where the OF medium is deployed and is considered as the most sensitive part of the network in terms of failures and maintenance. The analysis shows that the proposed ring-based architecture provides acceptable results in terms of connection availability for the feeder level OF medium. This improvement can be attributed to the fact that the OF medium employed at the feeder level of the proposed architecture is protected via a ring-based topology, which is capable of facilitating the flow of traffic in both clockwise and counterclockwise directions. Consequently, if a fault occurs at any point inside the ring, the switch at the switching arrangement moves from port 1 to 2 and restores the flow of traffic to the nodes beyond the point of failure. Thus, the proposed ring-based topology provides redundancy at the feeder level, which significantly elevates the overall availability of the network.

In contrast, the tree-based architecture utilizes a single feeder fiber to carry traffic from the CO toward the RN with no redundancy against the failures across the feeder fiber. Consequently, a single failure across the feeder fiber in the tree-based architecture can disrupt the flow of traffic between the CO and receiving nodes. Therefore, it can be observed that the connection availability of the fiber deployed across the feeder level is 99.997% for the tree-based architecture, which is significantly lower than the acceptable threshold of five nines.

The connection availability at the distribution level is observed in two steps. The first step observes the connection availability of the 2 km OF medium deployed among the adjacent RNs. The analysis indicates that a connection availability of 99.9994% is obtained for the optical fiber medium deployed between the adjacent RNs. This can be attributed to the fact that short-span OF media are deployed between adjacent RNs that results in the elevation of connection availability. Furthermore, it can be observed that each RN can be fed from both clockwise and counterclockwise directions. Therefore, a failure across the OF media deployed between RNs does not affect the connection availability for the ONTs.

For further analysis, DF deployed between the RNs and ONT modules is also considered for both networks. This is performed by considering 3 km and 5 km OF media between RNs of the proposed ring and tree-based architectures, respectively. It can be observed that the distribution of the OF media between the RNs and ONT modules for the proposed ring-based architecture has reduced the overall span of the fiber leading to the ONT modules. Consequently, a connection availability of 99.9992% is obtained for the optimized architecture, which is above the acceptable threshold of five nines. On the contrary, a connection availability of 99.998% is obtained for the tree-based architecture that deploys a 5 km DF between RNs and their corresponding ONTs.

Thus, it can be observed that optimization of the 2D spectral/spatial OCDMA architecture with ring-based topology has significantly elevated the connection availability at both the feeder and distribution levels.

## 6. Comparative Analysis

Table 6 compares the proposed 2D DW-ZCC ring-based OCDMA system with spectral–spatial and temporal encoding with existing systems based on system cardinality, data rate, received power, reliability, and encoding–decoding techniques. It can be observed that the proposed system supports a cardinality of 128, similar to its conventional 2D DW-ZCC counterpart but optimized for higher reliability and performance.

In terms of data rate, the proposed 2D DW-ZCC ring-based OCDMA system significantly outperforms conventional 3D implementations, like 3D diagonal eigenvalue unity and multi-diagonal (DEU-DEU-MD), 3D multi-diagonal (MD), 3D single weight zero cross-correlation (SW-ZCC), and 3D perfect difference/multi-diagonal (PD-MD), which operate in the range of 1 to 5 Gbps, while the proposed system reaches 10 Gbps. It is worth mentioning that most of the existing work provides analytical analysis of 3D OCDMA systems. Additionally, it can be observed that the proposed system operates efficiently at a received power of approximately −19 dBm, close to the 2D DW-ZCC system’s −21 dBm and considerably better than conventional 3D systems that require higher power levels, such as −10 dBm for a 3D SW-ZCC code-based system.

Furthermore, it can be observed that the proposed system is optimized to ensure high reliability and low-cost implementation unlike conventional 3D systems that are implemented with multiple parallel long-span optical fiber links for spatial encoding and decoding. Consequently, these systems require a high cost of implementation and offer no reliability against system failure at the feeder level. In summary, the proposed system offers superior data rates, better power tolerance, and greater reliability, making it a highly efficient solution for optical network communications.

## 7. Conclusions

The proposed fault-tolerant 2D spectral–spatial OCDMA-based PON with time delay demonstrates a robust and efficient solution for next-generation communication systems, such as 5G, 6G, and beyond. By employing a ring topology at the FL, the network effectively mitigates the single point of failure issue inherent in tree-based PONs, thereby enhancing connection availability without compromising transmission capacity. Utilizing a pseudo-3D double-weight zero cross-correlation code, the system achieves high cardinality and capacity, supporting up to 128 users at 10 Gbps with minimal BER, outperforming conventional systems like 3D-DEU-DEU-MD and 3D PD-MD in terms of data rate and received power. Optisystem simulations reveal that the optimized architecture maintains performance under both normal and fault conditions, with acceptable BER and QF values and clear eye diagrams even at OF lengths of up to 16 km and a received power of approximately −19 dBm, highlighting its improved power tolerance over competing systems. The network’s resilience is further strengthened by its 99.999% connection availability for the optical fiber media at both the feeder and distribution levels, unlike any of the existing OCDMA systems with spectral–spatial and temporal encoding in the existing literature. Thus, the optimized architecture not only supports high data rates and extensive subscriber reach but also provides desirable connection availability and reliability through a robust infrastructure, making it a superior candidate for future optical access networks.

## Figures and Tables

**Table 1:** The 2D DW-ZCC code.

$Y_{j}^{T}X_{i}$	$X_{0}\;=\;\left[1100\right]$	$X_{1}\;=\;\left[0011\right]$
$Y_{0}^{T}\left[\begin{array}{c} 1\\ 1\\ 0\\ 0 \end{array}\right]$	$\left[\begin{array}{c} 1100\\ 1100\\ 0000\\ 0000 \end{array}\right]$	$\left[\begin{array}{c} 0011\\ 0011\\ 0000\\ 0000 \end{array}\right]$
$Y_{1}^{T}\left[\begin{array}{c} 0\\ 0\\ 1\\ 1 \end{array}\right]$	$\left[\begin{array}{c} 0000\\ 0000\\ 1100\\ 1100 \end{array}\right]$	$\left[\begin{array}{c} 0000\\ 0000\\ 0011\\ 0011 \end{array}\right]$

**Table 2:** System performance parameters for simulation analysis.

System Parameter	Value
Signal power at CO	0 dBm
OF attenuation	0.25 dB/km
OF dispersion	18 ps/nm/km
Receiver’s responsitivity	0.85 A/W
User data	10 Gbits/s
Data format	NRZ
Thermal noise	$7.5\times 10^{-15}$
Distribution of noise	Gaussian
OF length (feeder level)	10 km
OF length (between RNs)	Variable

**Table 3:** BER, eye diagrams, and received power at each RN for clockwise flow of traffic.

RN	$RN_{1}$	$RN_{2}$	$RN_{3}$	$RN_{4}$
Eye Diagram	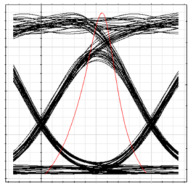	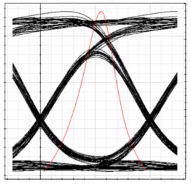	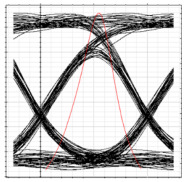	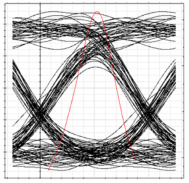
BER	$5.11{\times 10}^{-18}$	$3.2{\times 10}^{-16}\;$	$1.6{\times 10}^{-14}$	$8.3{\times 10}^{-11}$
Received Power	−9.6 dBm	−13 dBm	−16.2 dBm	−19.4 dBm

**Table 4:** BER and eye diagrams for clockwise and counterclockwise flow of traffic.

RN	$RN_{1}$	$RN_{2}$	$RN_{3}$	$RN_{4}$
Eye Diagram	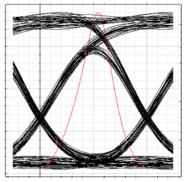	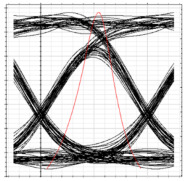	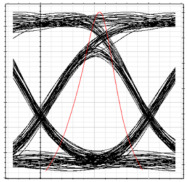	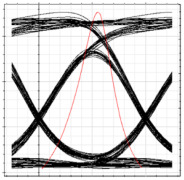
BER	$3.7{\;\times \;10}^{-18}$	$2.6{\;\times \;10}^{-15}$	$1.6{\;\times \;10}^{-14}$	$3.2{\;\times \;10}^{-16}$
Received Power	−9.8 dBm	−14.3 dBm	−16.2 dBm	−13 dBm

**Table 5:** Unavailability $\left(\frac{Failure}{10^{9}h}\right)$ for system components.

Component	Components Unavailability
Optical line terminal	$5.12\times 10^{-7}$
Optical coupler (1:2, 2:2)	$3\times 10^{-7}$
Optical coupler (1:N, 2:N)	$7.2\times 10^{-7}$
Time delay units	$4\times 10^{-7}$
Optical switch	$1.2\times 10^{-6}$
Mux/ De-Mux filters	$1\times 10^{-7}$
Optical network terminal	$5.12\times 10^{-7}$
OF/km	$2.8\times 10^{-6}$

**Table 6:** Comparison of the proposed architecture with existing systems.

Code Name	System Cardinality	Data Rate	Received Power (dBm)	Reliability	Encoding–Decoding
2D DW-ZCC [5]	128	10 Gbps	−21	None	Optimized
3D-DEU-DEU-MD [31]	136	1 Gbps	−13.9	None	Conventional
3D-MD [21]	100	1 Gbps	−12.8	None	Conventional
3D SW-ZCC [23]	200	4.8 Gbps	−10	None	Conventional
3D PD-MD [32]	150	1 Gbps	Not given	None	Conventional
Proposed	128	10 Gbps	−19	High	Optimized

## Data Availability

No new data were created or analyzed in this study.
